# Six-year time-trend analysis of dyslipidemia among adults in Newfoundland and Labrador: findings from the laboratory information system between 2009 and 2014

**DOI:** 10.1186/s12944-018-0752-2

**Published:** 2018-05-02

**Authors:** Pardis Pedram, Erfan Aref-Eshghi, Hensley H. Mariathas, Oliver Hurley, Marshall Godwin, Pauline Duke, Masoud Mahdavian, Shabnam Asghari

**Affiliations:** Faculty of Medicine, Memorial University of Newfoundland, Center for Rural Health Studies, Room M5M107, Health Sciences Centre, 300 Prince Philip Drive, St. John’s, NL A1B 3V6 Canada

**Keywords:** Dyslipidemia, Newfoundland, HDL-C, LDL-C, Cholesterol, Trend, Fixed effect, Random effect

## Abstract

**Background:**

Dyslipidemia, an increased level of total cholesterol (TC), triglycerides (TG), low-density-lipoprotein cholesterol (LDL-C) and decreased level of high-density-lipoprotein cholesterol (HDL-C), is one of the most important risk factors for cardiovascular disease. We examined the six-year trend of dyslipidemia in Newfoundland and Labrador (NL), a Canadian province with a historically high prevalence of dyslipidemia.

**Methods:**

A serial cross-sectional study on all of the laboratory lipid tests available from 2009 to 2014 was performed. Dyslipidemia for every lipid component was defined using the Canadian Guidelines for the Diagnosis and Treatment of Dyslipidemia. The annual dyslipidemia rates for each component of serum lipid was examined. A fixed and random effect model was applied to adjust for confounding variables (sex and age) and random effects (residual variation in dyslipidemia over the years and redundancies caused by individuals being tested multiple times during the study period).

**Results:**

Between 2009 and 2014, a total of 875,208 records (mean age: 56.9 ± 14.1, 47.6% males) containing a lipid profile were identified. The prevalence of HDL-C and LDL-C dyslipidemia significantly decreased during this period (HDL-C: 35.8% in 2009 [95% CI 35.5-36.1], to 29.0% in 2014 [95% CI: 28.8-29.2], *P* = 0.03, and LDL-C: 35.2% in 2009 [95% CI: 34.9-35.4] to 32.1% in 2014 [95% CI: 31.9-32.3], *P* = 0.02). A stratification by sex, revealed no significant trend for any lipid element in females; however, in men, the previously observed trends were intensified and a new decreasing trend in dyslipidemia of TC was appeared (TC: 34.1% [95% CI 33.7-34.5] to 32.3% [95%CI: 32.0-32.6], *p* < 0.02, HDL-C: 33.8% (95%CI: 33.3-34.2) to 24.0% (95% CI: 23.7-24.3)], *P* < 0.01, LDL-C: 32.9% (95%CI:32.5-33.3) to 28.6 (95%CI: 28.3-28.9), *P* < 0.001). Adjustment for confounding factors and removing the residual noise by modeling the random effects did not change the significance.

**Conclusion:**

This study demonstrates a significant downward trend in the prevalence of LDL-C, HDL-C, and TC dyslipidemia, exclusively in men. These trends could be the result of males being the primary target for cardiovascular risk management.

## Background

Cardiovascular disease (CVD), remains to be the first cause of death globally, with 17.7 million deaths each year [1]. In Canada, over 1.3 million people have a diagnosis of heart disease [2], and CVD is the leading cause of death (one-third of the total deaths) [3], imposing the highest economic burden of disease after musculoskeletal conditions [2]. In the past decades, significant improvements have been reported in the rates of CVD and its associated risk factors in the developed world. Over the past 50 years, there has been a 70% decline in the incidence of CVD in Canada [2]. Compared to the other Canadian provinces, however, Newfoundland and Labrador (NL) has the highest rates of CVD morbidity and mortality in adults [4]. In 2007, Statistics Canada reported that NL had the highest age-standardized mortality rate for major CVD events (218.5 per 100,000 population) among all Canadian provinces (national average mortality rate: 151.9/100,000) [5]. This rate is comparable to those of low- or middle-income nations which tend to have higher CVD-related death rates compared to the developed world [6, 7], and do not show a declining trend in CVD incidence [8].

The declining global trends in CVD incidence has been mainly attributed to decreasing rates of preventable CVD risk factors such as tobacco use [9], uncontrolled hypertension [10], and abnormal lipid levels [11]. Dyslipidemia, defined as abnormally elevated levels of total cholesterol (TC), low-density lipoprotein cholesterol (LDL-C), and triglycerides (TG), as well as decreased levels of high-density lipoprotein cholesterol (HDL-C), is one of the strongest and most modifiable risk factors for CVD [12]. Like other CVD risk factors, there has been an improvement in the abnormal cholesterol levels among Canadians in the past decade, and this trend is projected to continue over the next few years [11]. However, it is not known whether the declining trend in the prevalence of dyslipidemia has reached the NL population. Our previous study demonstrated that the prevalence of dyslipidemia, as represented by abnormal levels of total cholesterol (40 vs. 37%), LDL-C (29 vs. 25%), triglyceride (29 vs. 26%) and HDL-C (38 vs. 27%), is significantly higher in NL compared to the rest of Canada [13]. Although the cultural and genetic isolation of the NL population [14] might partially explain the differences in the lipid profiles, our study on high-cardiac risk patients has shown that primary care management, particularly medication therapy, is the most contributing factor to suboptimal lipid levels in Canada, and particularly, in NL [15]. Therefore, any change in the pattern of dyslipidemia could well reflect the quality of healthcare in the province, and thus, the higher rates of CVD and dyslipidemia prevalence in NL necessitates the investigation of the trend of the change in dyslipidemia.

The availability of Laboratory Information System (LIS) in NL, which enables access to all of the laboratory test results conducted in the province, provides an excellent opportunity to investigate the rates of dyslipidemia and its patterns over the past years. Discovery of this trend in NL is essential since it can be helpful for the estimation of future prevalence of dyslipidemia and its consequential burden on the health care system. Also, these data are necessary for evaluation and monitoring of the effectiveness of population- and community-wide interventions in reducing the prevalence of dyslipidemia and its adverse consequences in NL, and accordingly, in Canada. The present study examines the trend and pattern of dyslipidemia among the adult population living in NL over a six-year period between 2009 and 2014.

## Methods

### Source of data

Individuals with a registered permanent address in NL are provided with a lifetime medical care plan number. For each laboratory service, the patient’s identification (ID), date of service, and laboratory test results are entered into the LIS. The system is administered through four health region authorities in the province (Eastern, Central, Western, and Labrador-Grenfell). The study extracted all available records included in the LIS between January 2009 and December 2014. Every record contained individual de-identified ID, age, sex, date of testing, and lipid test results. Only records for individuals who were 20 years and older at the time of lipid testing, were used in this study.

### Identification of trends of change in dyslipidemia

We defined dyslipidemia based on lipid levels, according to the Canadian Guidelines for the Diagnosis and Treatment of Dyslipidemia (Table 1), in effect during the time of the study [16, 17]. The prevalence of dyslipidemia in each year was calculated as the ratio of records with abnormal levels of lipid to the total number of records available in that year. Descriptive statistics, including mean and 95% confidence intervals (CI), were used to describe and visualize the changes in the prevalence of dyslipidemia over the 6-year period. A non-parametric trend test was performed to examine the existence of a changing trend in dyslipidemia during this time.

**Table 1:** Healthy levels of serum lipids for Canadian adults [3]

Lipid component	Normal levels
Total cholesterol (TC)	< 5.2 mmol/L
Triglycerides (TG)	< 1.7 mmol/L
Low-density lipoprotein (LDL-C) cholesterol	< 3.4 mmol/L
High-density lipoprotein (HDL-C) cholesterol	Males > 1.0 mmol/L
	Females > 1.3 mmol/L

### Adjustment for confounding factors and control for repeated individual tests and random yearly variations

Assessment of the variability in the lipid profiles as reported by the four different regional health authorities was determined by measuring an intra-class correlation which did not reveal a significant clustering by health region (not shown). Therefore this variable was not used in this analysis. A multivariable logistic mixed effect model was utilized to adjust for the confounding variables including age and sex, as well as to account for variations caused by the same individuals repeating the tests over the 6-year period or the normal yearly changes. In this model, dyslipidemia status was considered as the response variable, the year was considered a categorical predictor, and age and sex were incorporated as confounding factors. The first year (2009) was considered as the base, to which all of the upcoming years were compared. Given an individual could be tested for lipid profile multiple times across the 6-year period, we incorporated the individual ID as a random effect in the model to account for this redundancy. As well, we incorporated the year variable as the second random effect to reduce the residual variation that typically occurs on a yearly basis in dyslipidemia rates. The analysis was conducted using the *lme4* package [18].

All of the statistical analyses were performed using R.3.3.0. *P*-values less than 0.05 were considered significant.

## Results

### Characteristics of the study population

After quality assessment and removal of missing information, a total of 875,208 records with lipid tests were identified in LIS, representing the total number of tests performed in the province between 2009 and 2014. A considerable gradual increase in the number of tests was noted from the first year (2009, ~ 115,000) to the last year of the study (2014, ~ 184,000). The population was almost equally composed of males and females with slightly more records identified for women (52 - 53% females). The mean age ranged from 56 to 58 during the 6-year period. Table 2 represents the characteristics of the study population as stratified by each year included in our study.

**Table 2:** Trends in dyslipidemia in Newfoundland and Labrador adults 2009-2014

	2009	2010	2011	2012	2013	2014	*P*-value
Record count (% Males)	115,382 (48%)	134,710 (47%)	134,832 (47%)	143,322 (47%)	164,734 (48%)	182,228 (48%)	
Age	56.0 (55.9-56.1)	55.8 (55.7-55.8)	56.2 (56.1-56.3)	56.5 (56.5-56.6)	57.7 (57.6-57.7)	58.3 (58.2-58.3)	
TC dyslipidemia							
Total	39.0 (38.7-39.3)	39.0 (38.7-39.3)	38.6 (38.3-38.9)	37.5 (37.2-37.7)	37.0 (36.7-37.2)	38.2 (38.0-38.4)	0.1
Female	43.5 (43.1-43.9)	43.2 (42.9-43.6)	43.3 (42.9-43.6)	42.2 (41.9-42.6)	41.7 (41.4-42.0)	43.6 (43.3-43.9)	0.5
Male	34.1 (33.7-34.5)	34.2 (33.9-34.6)	33.4 (33.1-33.8)	32.2 (31.9-32.6)	31.8 (31.5-32.1)	32.3 (32.0-32.6)	0.02*
HDL-C dyslipidemia							
Total	35.8 (35.5-36.1)	32.6 (32.4-32.9)	30.3 (30.0-30.6)	32.8 (32.6-33.1)	30.0 (29.8-30.3)	29.0 (28.8-29.2)	0.03*
Female	37.5 (37.1-37.9)	35.2 (3.8-35.6)	33.7 (33.4-34.1)	36.6 (36.2-36.9)	34.6 (34.3-34.9)	33.4 (33.1-33.7)	0.2
Male	33.8 (33.3-34.2)	29.6 (29.2-30.0)	26.3 (26.0-26.7)	28.5 (28.1-28.8)	24.8 (24.5-25.1)	24.0 (23.7-24.3)	0.01*
LDL-C dyslipidemia							
Total	35.2 (34.9-35.4)	33.7 (33.5-34.0)	32.5 (32.2-32.7)	32.5 (32.3-32.8)	31.2 (31.0-31.4)	32.1 (31.9-32.3)	0.02*
Female	37.2 (36.8-37.6)	35.5 (35.1-35.9)	34.5 (34.1-34.9)	35.1 (34.7-35.4)	33.7 (33.4-34.0)	35.3 (34.9-35.6)	0.2
Male	32.9 (32.5-33.3)	31.7 (31.3-32.1)	30.2 (29.9-30.6)	29.7 (29.3-30.1)	28.5 (28.1-28.8)	28.6 (28.3-28.9)	0.001*
TG dyslipidemia							
Total	36.4 (36.1-36.6)	35.0 (34.8-35.3)	36.3 (36.1-36.6)	36.2 (35.9-36.4)	35.6 (35.3-35.8)	35.9 (35.6-36.1)	0.8
Female	34.1 (33.8-34.5)	32.9 (32.5-33.2)	34.1 (33.8-34.5)	34.0 (33.6-34.3)	33.6 (33.2-33.9)	34.1 (33.8-34.4)	0.7
Male	38.8 (38.4-39.2)	37.4 (37.0-37.8)	38.8 (38.4-39.2)	38.7 (38.3-39.1)	37.8 (37.4-38.1)	37.7 (37.4-38.1)	0.5

Age is presented as mean (95% confidence interval), and dyslipidemia is presented as Percentage (95% confidence interval). *TC* Total cholesterol, *HDL-C* High-density lipoprotein cholesterol, *LDL-C* Low-density lipoprotein cholesterol, *TG* Triglycerides

*Trend is significant at *p* < 0.05

### Changes in the pattern of dyslipidemia between 2009 and 2014

**Fig. 1:**
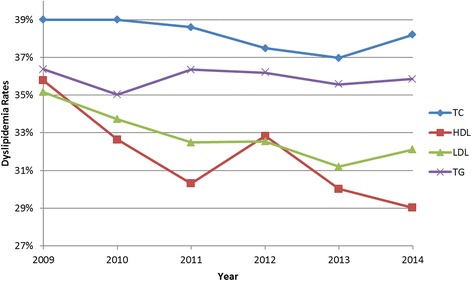
Rates of dyslipidemia by lipid components over six years: TC: Total cholesterol; HDL-C: High-density lipoprotein cholesterol, LDL-C: Low-density lipoprotein cholesterol, TG: Triglycerides

Examination of the rates of dyslipidemia over the 6-year period revealed a declining trend for HDL-C (35.8% [95% confidence interval: 28.8 - 29.2] to 29.0% [95% confidence interval: 28.8 - 29.2], *p* = 0.03) and LDL-C (35.2% [95% confidence interval: 34.9 - 35.4] to 32.1% [95% confidence interval: 31.9 - 32.3], *p* = 0.02). Other lipid components fluctuated during the same period without overall significant changes. Total cholesterol dyslipidemia, for instance, showed a decreasing trend from 39% to 37% in 2013, but its rates increased to above 38% in 2014 (Fig. 1, Table 2). Re-examination of the trends following a split by sex, however, revealed that the declining pattern of change in HDL-C and LDL-C dyslipidemia is only significant in males. The split also resulted in the appearance of a significant decreasing trend in total cholesterol dyslipidemia in males (34.1% [95% confidence interval: 33.7 - 34.5] to 32.3% [95% confidence interval: 32.0 - 32.6], *p* = 0.02), but not in females (Table 2).

### Trend of dyslipidemia after adjustment for confounding factors and random effects

Using a multivariable logistic mixed effect model, we examined the yearly pattern of change as adjusted for confounding factors (age and sex) and random effects (individuals repeating the test in the 6-year period, and regular annual variations in dyslipidemia). The analysis confirmed the previously observed declining rates of dyslipidemia over the 6-year period with the most prominent effect for HDL-C and LDL-C dyslipidemia. According to this analysis, the odds of finding an HDL-C or LDL-C dyslipidemia in the laboratory test reports in the last year of the study was significantly lower than those in the first year (OR_HDL-C_: 0.78, 95% confidence interval: 0.77 - 0.79, OR_LDL-C_: 0.87, 95% confidence interval: 0.85 - 0.88). Although a declining trend was observed for other lipid components, these findings were not as prominent as those found for LDL-C and HDL-C, with odds ratios ranging between 0.95 to 0.99. The most determinant variable of dyslipidemia in the population, however, was sex where males were significantly less likely to have dyslipidemia of HDL-C, LDL-C, and total cholesterol (OR: 0.60 – 0.78), but more likely to have a dyslipidemia of triglyceride (OR: 1.27, 95% confidence interval: 1.26 - 1.28). No effect was observed for age. The result of this analysis is presented in Table 3.

**Table 3:** Risk of dyslipidemia over the five years after 2009 using a mixed effect model

Variable	TC	HDL-C	LDL-C	TG
Year				
2010	0.97 (0.95-0.99)	0.92 (0.90-0.94)	0.96 (0.94-0.98)	0.92 (0.90-0.94)
2011	0.95 (0.93-0.97)	0.84 (0.83-0.86)	0.87 (0.85-0.88)	0.98 (0.96-1.00)
2012	0.90 (0.88-0.91)	0.94 (0.92-0.95)	0.88 (0.87-0.90)	0.97 (0.95-0.99)
2013	0.89 (0.88-0.91)	0.83 (0.81-0.84)	0.85 (0.83-0.86)	0.94 (0.92-0.95)
2014	0.96 (0.94-0.98)	0.78 (0.77-0.79)	0.87 (0.85-0.88)	0.95 (0.93-0.97)
Sex (M)	0.60 (0.59-0.60)	0.66 (0.65-0.66)	0.78 (0.77-0.79)	1.27 (1.26-1.28)
Age	0.99 (0.99-0.99)	1.00 (1.00-1.00)	0.98 (0.98-0.98)	1.00 (1.00-1.00)

*TC* Total cholesterol, *HDL-C* High-density lipoprotein cholesterol, *LDL-C* Low-density lipoprotein cholesterol, *TG* Triglycerides. Figures are odds ratio (95% Confidence Intervals). Every year is compared to 2009 as the base. Year and individual identifier are incorporated as random effects in the model

## Discussion

The current study described the trend of dyslipidemia over a six-year period during 2009 to 2014 in NL using provincial medical laboratory information. The analyses identified a significant downward trend in the prevalence of dyslipidemia of LDL-C and HDL-C during this period. Further evaluations indicated that this declining pattern is mainly represented in males, whereas females do not show a significant change in the trend of dyslipidemia. Being male was also associated with a lower risk of having any form of dyslipidemia except for triglyceride.

Dyslipidemia is a highly manageable risk factor for CVD. The association between hyperlipidemia and the incidence, morbidity, and mortality of CVD has been reported in numerous studies [19, 20]. Every 1.0 mmol/L (38.6 mg/dl) reduction in LDL-C is found to be associated with a ~ 23% relative risk reduction in major vascular events over a five-year period [21]. Similarly, every 1.0 mg/dl increase in HDL-C has resulted in a 2-3% reduction in the occurrence of coronary heart disease [22]. Guidelines for the management of lipid levels as a means of CVD risk reduction have been in effect for decades, and these practices have resulted in a significant decrease in the rates of dyslipidemia, and subsequently CVD, in the developed world. Examination of a 30-year trend (1976-2006) in serum lipids among adults in the United States has shown decreases in age-adjusted mean TC (210 to 200 mg/dl) and LDL-C (134 to 119 mg/dl) as well as a significant increase in mean HDL-C (50 to 53 mg/dl) [23]. Similar patterns of change have been observed in Europe, as reported in a 10-year period by the Framingham Heart Study [24], and the northern Sweden MONICA study [25]. In Canada, projected rates of high cholesterol, based on the observed rates from the Canadian Health Measure Survey, indicates a gradual decrease in hypercholesterolemia between 2001 and 2021 [11].

In NL, however, no data is available on the time trend of change in dyslipidemia, besides the few recent reports of a higher rate of hypercholesterolemia compared to the rest of Canada. Our study is the first to examine such a trend over a six-year period in NL. Approximately ten years ago, a survey of waist circumference and body mass index in NL recommended that young NL adults are at an elevated risk of cardiovascular events due to the distribution of body fat [26]. Since then, multiple evaluations by our group and others recommended that the NL population may also suffer from a higher rate of dyslipidemia and other CVD risk factors [13]. These reports could have led to an increased awareness among the clinicians in NL. The findings in the present study recommend that NL is at the beginning of a movement toward improving cardiometabolic profiles. Our results support a declining trend in the rates of dyslipidemia of HDL-C, LDL-C, and TC. However, the level of change is not as significant as those found elsewhere, and any degree of improvement is restricted to men. These may be an indication of a recent movement towards the improvement of primary care management of dyslipidemia in the province.

An interesting finding in this study was that any declining trend in the rates of abnormal lipid profiles is restricted to men. Also, being male was significantly associated with a lower risk of having any dyslipidemia, except for TG. These might suggest that men have been targeted more intensively for CVD risk management. An alternative explanation might be related to the distribution of the age of our population. At the mean age of > 55, women are influenced by the physiological changes of menopause and are likely to show a higher rate of dyslipidemia [27]. It is possible that due to these physiological changes, women are not as responsive to CVD risk interventions as men [28, 29]. These hypotheses, however, will need to be further investigated.

Our study is limited by a few factors which need to be taken into consideration. The findings are built on the clinical laboratory tests conducted in the province and thus may not show a crude prevalence of dyslipidemia in NL. However, the results represent any individual who has ever performed a lipid test in the province during the six-year period, and thus, they can be used as a reliable indicator of population health as related to CVD and dyslipidemia. The other limitation of this study is the lack of information on the confounding factors that might influence dyslipidemia (e.g., body mass index, diabetes, menopause status and smoking). Also, the information related to lipid modifying agent consumption was not available. Lifestyle-related factors related to hypercholesterolemia such as nutritional behaviors, physical activity and awareness towards primary prevention were not included in this study since we did not have access to datasets where these variables were included.

## Conclusion

The findings of this study reveal that NL has started to follow the declining trend in the rates of dyslipidemia. This is mainly represented as a significant downward trend in the rates of LDL-C, HDL-C, and TC dyslipidemia in men. These trends could be the result of males being the primary target for cardiovascular risk management guidelines; however, further research is required to verify this hypothesis. The province of Newfoundland and Labrador could see a reduction in cardiovascular disease incidence along with their associated mortality rates following an improvement in lipid profiles within the next few years. Our study’s findings indicate the urgency for further investigation into this matter which can be considered essential for the proper surveillance of chronic disease outcomes in the province.

## Abbreviations

CVD: Cardiovascular disease
HDL-C: High-density lipoprotein cholesterol
LDL-C: Low-density lipoprotein cholesterol
LIS: Laboratory information system
NL: Newfoundland and Labrador
OR: Odds ratio
TC: Total cholesterol
TG: Triglyceride
